# Milestone Review: Unlocking the Proteomics of Glycine Receptor Complexes

**DOI:** 10.1111/jnc.70061

**Published:** 2025-04-25

**Authors:** Sean D. Fraser, Remco V. Klaassen, Carmen Villmann, August B. Smit, Robert J. Harvey

**Affiliations:** ^1^ School of Health University of the Sunshine Coast Maroochydore Queensland Australia; ^2^ National PTSD Research Centre Thompson Institute, University of the Sunshine Coast Birtinya Queensland Australia; ^3^ Department of Molecular and Cellular Neurobiology, Center for Neurogenomics and Cognitive Research Amsterdam Neuroscience, Vrije Universiteit Amsterdam Amsterdam the Netherlands; ^4^ Institute of Clinical Neurobiology University Hospital, Julius‐Maximilians‐University of Würzburg Würzburg Germany

**Keywords:** Collybistin, gephyrin, glycine receptor, proteomics, startle disease, syndapin I

## Abstract

Glycine receptors (GlyRs) are typically known for mediating inhibitory synaptic transmission within the spinal cord and brainstem, but they also have key roles in embryonic brain development, learning/memory, inflammatory pain sensitization, and rhythmic breathing. GlyR dysfunction has been implicated in multiple neurological disease states, including startle disease (GlyR α1β) and neurodevelopmental disorders (NDDs) including autism spectrum disorder (ASD), intellectual disability (ID), developmental delay (DD) and epilepsy (GlyR α2). However, GlyRs do not operate in isolation but depend upon stable and transient protein–protein interactions (PPIs) that influence synaptic localization, homeostasis, signaling pathways, and receptor function. Despite the affinity purification of GlyRs using the antagonist strychnine over four decades ago, we still have much to learn about native GlyR stoichiometry and accessory proteins. In contrast to other neurotransmitter receptors, < 20 potential GlyR interactors have been identified to date. These include some well‐known proteins that are vital to inhibitory synapse function, such as the postsynaptic scaffolding protein gephyrin and the RhoGEF collybistin. However, the majority of known interactors either bind to the GlyR α1 and β subunits, or the binding partner in the GlyR complex is unknown. Several potential GlyR interactors are not found at inhibitory synapses and/or have no clear functional role. Moreover, other GlyR interactors are *secondary interactors* that bind indirectly, for example, via gephyrin. In this review, we provide a critical evaluation of known GlyR interacting proteins and methodological limitations to date. We also provide a road map for the use of innovative and emerging interaction proteomic techniques that will unlock the GlyR interactome. With the emergence of disease‐associated missense mutations in the α1, α2 and β subunit intracellular domains in startle disease and NDDs, understanding the identity and roles of GlyR accessory proteins is vital in understanding GlyR function and dysfunction in health and disease.

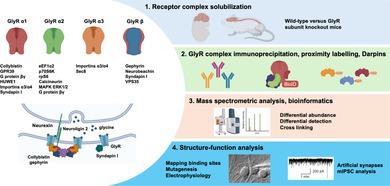

## Graphical abstract

Glycine receptors (GlyRs) play key roles in brain development, learning/memory, inflammatory pain sensitization, and rhythmic breathing. GlyRs depend upon stable and transient protein–protein interactions that influence synaptic localization, homeostasis, signaling pathways, and receptor function. However, since they were first affinity purified over four decades ago, remarkably few synaptic GlyR interactors have been discovered. We provide a critical evaluation of known GlyR interactors and a road map for the use of innovative interaction proteomic techniques that will unlock the GlyR interactome and provide insights into disease mechanisms in startle disease and neurodevelopmental disorders.

**Abbreviations**
ArfGEF: guanine nucleotide exchange factor for Arf GTPases
GABA_A_R: γ‐aminobutyric acid type A receptor
GlyR: glycine receptor
PH: pleckstrin homology domain
PPIs: protein–protein interactions
RhoGEF: guanine nucleotide exchange factor for Rho/Rac/Cdc42‐like GTPases
SH3: *src* homology 3 domain
SynGO: synaptic gene ontologies

## 1 Introduction

Glycine receptors (GlyRs) belong to the family of pentameric Cys‐loop ligand‐gated ion channels, which also includes the nicotinic acetylcholine receptor (nAChR), serotonin type 3 receptor (5‐HT_3_R), and γ‐aminobutyric acid type A receptor (GABA_A_R) (Lynch 2004). Initially affinity‐purified from the rat and pig spinal cord in the early 1980s (Pfeiffer et al. 1982; Graham et al. 1985) by amino strychnine affinity chromatography, GlyR preparations contained three major proteins of molecular weights 48 kDa, 58 kDa, and 93 kDa. Molecular cloning revealed that the 48 kDa and 58 kDa components represent the GlyR α1 and β subunits (Grenningloh et al. 1987; Grenningloh, Pribilla et al. 1990), while the 93 kDa component is a synaptic scaffolding protein known as gephyrin (from the Greek *gephyra*, for bridge; Prior et al. 1992). GlyRs consist of a pentameric assembly of membrane‐spanning subunits symmetrically arranged around a central, ion‐conducting pore (Du et al. 2015; Zhu and Gouaux 2021). Both homopentameric α or heteromeric αβ receptors can be formed in vitro, which differ in pharmacology and ion‐channel conductance states (Pribilla et al. 1992; Bormann et al. 1993). The stoichiometric configuration of native GlyRs has been a matter of debate. However, recent studies have suggested that native heteromeric GlyRs have an invariant 4α:1β configuration (Yu et al. 2021; Zhu and Gouaux 2021). Each GlyR subunit comprises an N‐terminal signal peptide (SP), an agonist‐binding extracellular domain (ECD), four transmembrane domains (TM1‐TM4) an intracellular domain (ICD) between TM3‐TM4, and a short extracellular C‐terminus (Du et al. 2015; Yu et al. 2021; Zhu and Gouaux 2021). Five distinct GlyR subunits have been described in humans and rodents: the GlyR α1‐α4 and β subunits. The GlyR α1‐α4 subunits exhibit a high degree of sequence identity (≥ 80%; Grenningloh, Schmieden, et al. 1990; Kuhse et al. 1990; Matzenbach et al. 1994) which hampered the design of subunit‐specific antibodies for many years. The region featuring the least sequence similarity in GlyR α1‐α4 and β subunits is the TM3‐TM4 intracellular domain (ICD), which provides sites for interactions with accessory proteins and subunit‐specific modulation by ubiquitination (Büttner et al. 2001) or phosphorylation by protein kinases (Ruiz‐Gómez et al. 1991; Harvey, Depner et al. 2004; Manzke et al. 2010).

GlyRs containing the α1 and β subunits GlyR (α1β) are highly expressed in the spinal cord and brainstem, and consistent with this, several spontaneous mouse mutants in the corresponding genes (*Glra1* or *Glrb*) exhibit neuromotor defects and develop an exaggerated startle response to acoustic or tactile stimuli at around 2 weeks of age (Schaefer et al. 2013). Accordingly, GlyR α1β dysfunction causes startle disease/hyperekplexia, characterized by exaggerated startle reflexes in response to unexpected stimuli, muscle hypertonia, and neonatal apnea episodes (Shiang et al. 1993; Rees et al. 2002; Schaefer et al. 2022). The development of GlyR α2 knockout mice established a major role for GlyR α2 in cortical interneuron migration (Avila et al. 2013, 2014). Genetic disruption of GlyR α2 disrupted cortical progenitor homeostasis, impairing the capacity of apical progenitors to generate basal progenitors, resulting in an overall reduction of projection neurons in the cerebral cortex (Avila et al. 2013, 2014). As a result, microcephaly was observed in newborn *Glra2* knockout mice (Avila et al. 2014), as well as increased susceptibility to seizures (Morelli et al. 2017) and learning and memory defects (Pilorge et al. 2016; Molchanova et al. 2017). Consistent with these findings, missense mutations and deletions affecting the GlyR α2 subunit have been implicated in a range of neurodevelopmental disorders (NDDs) featuring autism spectrum disorder (ASD), developmental delay (DD), intellectual disability (ID) often accompanied by microcephaly, language delay, and epilepsy (Pilorge et al. 2016; Zhang et al. 2017; Chen et al. 2022; Marcogliese et al. 2022). GlyR α3 knockout and knock‐in mice have revealed roles for this subtype in central inflammatory pain sensitization (Harvey, Depner et al. 2004; Werynska et al. 2021) and rhythmic breathing (Manzke et al. 2010). Lastly, GlyR α4 is vital for correct embryonic development, and knockout mice have reduced litter sizes, impaired startle responses, and changes in social and anxiety‐like behaviors (Nishizono et al. 2020; Darwish et al. 2023).

**FIGURE 1:**
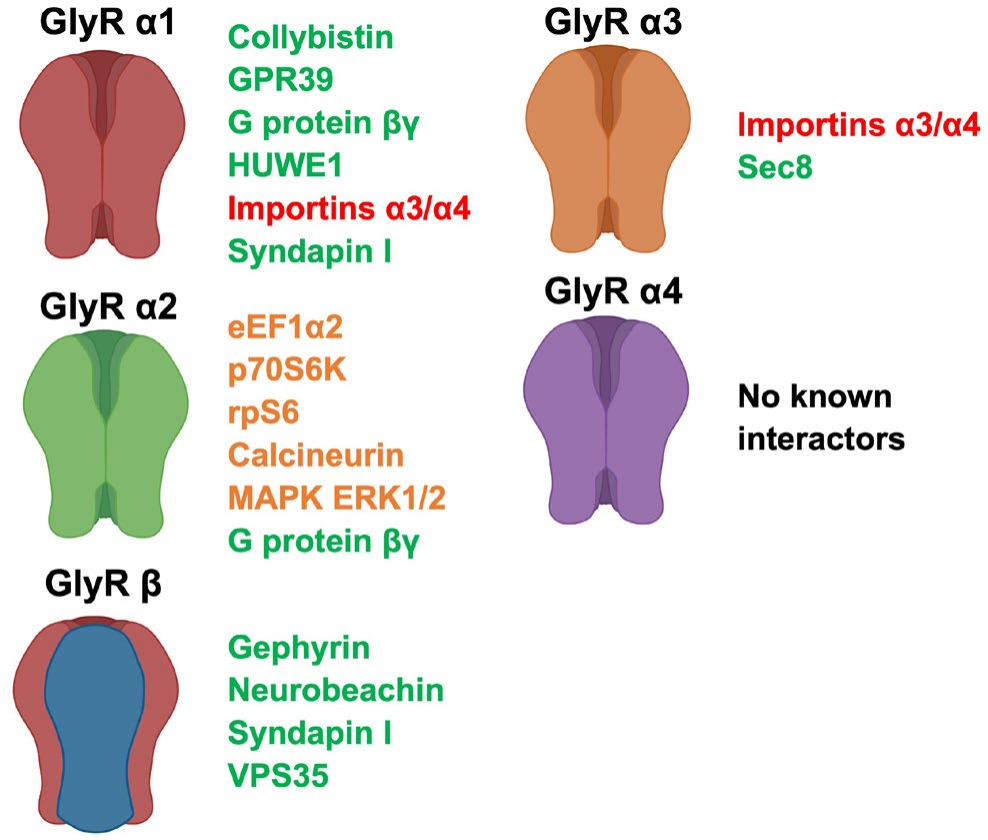
Glycine receptor subtypes and interactors. The traffic light coloring system indicates confidence in the interacting proteins based on available evidence: Green: Interactor with extensive supporting evidence for functional relevance; Amber: Interactor identified with a single method, with limited or unknown functional relevance; Red: Potential artifactual interactor. See also Table 1.

Despite recent advances in understanding the structure, biological roles, and molecular genetics of GlyRs, *critical knowledge gaps exist in our knowledge of GlyR interacting proteins*. For example, proteomic studies of inhibitory GABA_A_Rs have revealed hundreds of direct or indirect interaction partners that modulate receptor biosynthesis, trafficking, and function (Nakamura et al. 2016; Uezu et al. 2016; Chen, Koopmans, Paliukhovich, et al. 2023; Chen et al. 2023). By contrast, < 20 potential GlyR interactors have been identified to date (Figure 1; Table 1). These include some well‐known proteins that are localized at inhibitory synapses, including the scaffolding protein gephyrin (Prior et al. 1992), the RhoGEF collybistin (Kins et al. 2000; Grosskreutz et al. 2001; Harvey, Duguid et al. 2004; Poulopoulos et al. 2009; Saiepour et al. 2010; Breitinger et al. 2021), syndapin I/pacsin 1 (Del Pino et al. 2014; Langlhofer et al. 2020; Tröger et al. 2022), neurobeachin (Del Pino et al. 2011), glypican‐1 (van der Spek et al. 2020), neurexin 3 (van der Spek et al. 2020), the E3 ubiquitin ligase HUWE1 (Zhang et al. 2019) and the G‐protein‐coupled receptor GPR39 (Bai et al. 2024). Others have likely roles in GlyR trafficking, for example, vacuolar protein sorting ortholog 35 (VPS35; Del Pino et al. 2011) and the vesicular trafficking protein SEC8 (Winkelmann et al. 2014). However, the majority of known interactors appear to interact with the GlyR α1 or β subunits due to experimental bias towards this subtype. Moreover, other GlyR interactors are clearly *secondary interactors* that bind indirectly via gephyrin (e.g., the ArfGEF IQSEC3, Um et al. 2016; van der Spek et al. 2020), or the binding partner in the GlyR complex is unknown (e.g., glypican‐1, neurexin 3). Several other potential GlyR interactors have no clear functional role or are not found at inhibitory synapses. These include elongation factor eEF1α2 (Bluem et al. 2007), ribosomal proteins rpS6 and p70 ribosomal protein S6 kinase (Bluem et al. 2007), and importins α3/α4 that are involved in nuclear‐cytoplasmic transport (Melzer et al. 2010). This imperfect data set likely reflects a minority of true GlyR interactors in a mix of artifactual interactors that have been inadvertently identified in immunoprecipitation, GST‐fusion protein pulldowns, yeast two‐hybrid, or other proteomic assays. This review aims to summarize our current understanding of the GlyR interactome, including an analysis of potential experimental bias. We also provide a road map for using innovative and emerging interaction proteomic techniques to enable the identification, validation, and functional characterization of physiologically relevant GlyR accessory proteins.

**TABLE 1:** Binding partners and evidence for known GlyR interacting proteins.

GlyR	Interactor	Role/evidence/interaction partner(s)	Rating
α1	Collybistin	*Role*: Collybistin is a RhoGEF for the small GTPase Cdc42, which interacts with gephyrin, neuroligin 2, neuroligin 4, the small GTPase TC10 and the GABA_A_R α2 subunit. First identified as a gephyrin interactor responsible for synaptic clustering of gephyrin, collybistin was identified in GST‐GlyR α1 pulldowns using recombinant proteins. *Evidence*: Electrophysiology; GST pulldowns; mutagenesis. *Interaction*: Direct via GlyR α1 ICD motif ^365^PPPAPSKSP^373^ with the collybistin PH domain. Interactions can also be indirect via GlyR β‐gephyrin‐collybistin interactions. *Functional relevance*: Collybistin is vital for the synaptic localization of gephyrin and is regulated by multiple PPIs that relieve autoinhibition and induce a phospholipid affinity switch. *Key reference*: Breitinger et al. (2021)	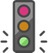
α1	G‐protein‐coupled receptor 39 (GPR39)	*Role*: GPR39 is an ‘orphan’ GPCR that signals via Gq, Gs, G12/13, Gi, and β‐arrestin. GPR39 has constitutive activity, and candidate ligands are Zn^2+^ and/or eicosanoids (15‐HETE and 14,15‐EET). GPR39‐GlyR α1 interactions were detected using a proximity ligation assay (PLA). GST‐GlyR α1 TM3‐TM4 ICD (but not GlyR α2 or β) purified FLAG‐tagged GPR39 overexpressed in cells. Targeted knockdown of GPR39 led to GlyR α1 tyrosine dephosphorylation at Y339 and reduced glycinergic inhibition. *Evidence*: PLA; GST pulldowns; mutagenesis; phosphospecific antibodies; *Gpr39* knockout mice. *Interaction*: GlyR α1 ICD binds to the intracellular C‐terminus of GPR39. *Functional relevance*: Modulation of GlyR α1 by tyrosine phosphorylation. *Key reference*: Bai et al. (2024)	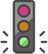
α1	HECT, UBA and WWE domain containing E3 ubiquitin‐protein ligase (HUWE1)	*Role*: HUWE1 is a E3 ubiquitin‐protein ligase that mediates ubiquitination and subsequent proteasomal degradation of target proteins. HUWE1 co‐localizes with gephyrin at inhibitory synapses. HUWE1‐GlyR α1 interactions were detected using IP with a HUWE1 antibody and subsequent Western blotting with GlyR antibodies. Recombinant HUWE1 enhanced the ubiquitination of GlyR α1, but not GlyR α3. HUWE1 knockdown in primary neurons via shRNA resulted in larger GlyR mIPSCs relative to control shRNAs. *Evidence*: IB; IP; ubiquitination assays; shRNA knockdown; electrophysiology. *Interaction*: GlyR α1 ICD. *Functional relevance*: GlyR ubiquitination, internalization and degradation. *Key reference*: Zhang et al. (2019)	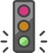
α1/α2	G protein βγ	*Role*: G‐protein βγ subunits bind to positively‐charged motifs in the GlyR α1 and GlyR α2 ICDs and for GlyR α1 cause an increase in GlyR open probability. *Evidence*: Electrophysiology; GlyR chimera analysis; Gβγ scavengers; GST fusion proteins; knock‐in mice; mutagenesis. *Interaction*: Direct via ICD motifs GlyR α1 ^316^RFRRK^320^ and ^385^KK^386^; GlyR α2 ^316^RFRRK^320^ and ^389^KR^390^. *Functional relevance*: Knock‐in mice containing mutations α1^K385A/K386A^ and GlyR α2^K389A/R390A^ that disrupt Gβγ interactions have reduced ethanol sensitivity, faster recovery from a sedative dose of ethanol, and higher ethanol intake. *Key references*: Yevenes et al. (2006, 2008, 2010); Aguayo et al. (2014); Gallegos et al. (2021); Muñoz et al. (2021)	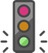
α1/α3	Importins α3/α4	*Role*: Importin α/β dimers mediate the translocation of proteins with nuclear localization signals (NLS) into the nucleus via nuclear pore complexes. GlyR α1/α3 ICDs contain NLS‐like sequences that interact with importins α3/α4 in yeast two‐hybrid assays using GlyR α1/α3 TM3‐TM4 ICDs as baits. eGFP‐tagged GlyR α1/α3 TM3‐TM4 ICD fusion proteins also show nuclear localization. For the eGFP‐GlyR α1 ICD fusion protein, mutation of ^318^RRKRR^322^ to alanine resulted in cytosolic location. *Evidence*: Nuclear import assays for eGFP‐ICDs; mutagenesis; YTH. *Interaction*: Direct via NLS‐like sequences in GlyR α1 (^318^RRKRR^322^) and α3 (^318^RRKRK^322^) ICDs, but these PPIs may be artifactual with no in vivo relevance. *Functional relevance*: unknown. *Key reference*: Melzer et al. (2010)	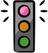
α1/β	Syndapin I	*Role*: Syndapin I (synaptic dynamin‐associated protein 1) is a synaptically‐enriched membrane tubulating protein implicated in membrane remodeling, endosomal/vesicle trafficking and Actin cytoskeletal dynamics. Syndapin I co‐IPed with native GlyRs from brainstem extracts, and the binding sites were mapped to the syndapin I SH3 domain and GlyR β ICD residues ^427^GKPQAKNKKPPPAKPVIPTAAK^448^. Binding was shown to be dependent on residues K434/K435 and P438/P441. Punctate syndapin I immunoreactivity was also demonstrated at glycinergic, GABAergic and mixed glycinergic/GABAergic synapses in cultured embryonic rat spinal cord neurons. Syndapin I knockdown resulted in a significant reduction in postsynaptic GlyR cluster size and number. Syndapin also interacts with GlyR α1 via a PXXPXR/K motif ^364^NNNNTTNPPPAPSKS^372^ and a startle disease‐associated missense variant GlyR α1^P366L^ reduced syndapin I binding in peptide microarray assays, but not GST‐fusion protein pulldowns or label‐free quantitative MS/MS. *Evidence*: GST pulldowns; peptide arrays; IB; IP; quantitative MS–MS; electrophysiology; artificial synapses. *Interaction*: Glyr α1 ICD, GlyR β ICD. *Functional relevance*: Syndapin I regulates postsynaptic GlyR cluster size, number, dispersal and mobility. *Key references*: Del Pino et al. (2014); Langlhofer et al. (2020); Tröger et al. (2022)	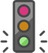
α2	Eukaryotic elongation factor 1 α2 (eEF1α2)	*Role*: eEF1α2 Is a GTPase that forms a complex with the guanine nucleotide exchange factor eEF1β2. eEF1α2 mediates mRNA translation by delivering aminoacyl‐tRNAs to ribosomes, but also binds and bundles Actin. eEF1α2 was identified in GST‐GlyR α2 pulldowns from rat brain extracts using MS. eEF1A immunoreactivity partially co‐localized with GlyRs in cultured neurons and redistributed from synapses and submembrane dendritic compartments to microtubules upon: (i) NMDAR stimulation; (ii) inhibition of GlyRs and stimulation of NMDARs or (iii) MAPK1/MAPK3 inhibition for extended culture periods. *Evidence*: IB; IC; GST pulldowns; MS; ICD. *Functional relevance*: Unknown, role in protein synthesis, regulation of microtubule dynamics, activity‐dependent redistribution of eEF1α2 during synapse maturation? *Key reference*: Bluem et al. (2007)	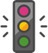
α2	p70 ribosomal protein S6 kinase (p70S6K)	*Role*: p70 ribosomal protein S6 kinase (p70S6K) is a serine/threonine kinase regulated by the phosphoinositide 3‐kinase (PI3K)/mammalian target of rapamycin (mTOR) signaling pathway and is known to phosphorylate ribosomal protein S6 (rpS6). p70S6K was identified in GST‐GlyR α2 pulldowns from rat brain extracts using MS. *Evidence*: GST pulldowns; IB; MS; phosphospecific antibodies. *Interaction partner*: GlyR α2 ICD. *Functional relevance*: Unknown. Glycine application to cultured neurones did not increase phosphorylation of p70S6K at position T389, which is known to increase p70S6K activity. *Key reference*: Bluem et al. (2007)	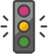
α2	Ribosomal protein S6 (rpS6)	*Role*: Ribosomal protein S6 (rpS6) is a component of the 40S ribosomal subunit. rpS6 was identified in GST‐GlyR α2 pulldowns from rat brain extracts using MS. Glycine application to cultured spinal cord neurons resulted in increased phosphorylation of rpS6. Immunocytochemistry demonstrated rpS6 in the soma, dendrites, and synapses in cultured hippocampal and spinal cord neurons, and rpS6 had partial overlap with GlyR immunoreactivity. *Evidence*: GST pulldowns; IB; IC; MS; phosphospecific antibodies. *Interaction partner*: GlyR α2 ICD. *Functional relevance*: Unknown, phospho‐rpS6 may control the local translation of a subset of mRNAs at active synapses? *Key reference*: Bluem et al. (2007)	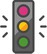
α2	Calcineurin	*Role*: Calcineurin is a Ca^2+^ and calmodulin‐dependent serine/threonine protein phosphatase (also known as protein phosphatase 3) formed from a calmodulin‐binding catalytic subunit calcineurin A (61 kDa) and a Ca^2+^‐binding regulatory subunit calcineurin B (19 kDa). A 58 kDa calcineurin isoform was identified in GST‐GlyR α2 pulldowns from rat brain extracts using MS, and confirmed using immunoblotting, but the specific isozyme of calcineurin A (PPP3CA, PPP3CB, PPP3CC) was not identified. *Evidence*: GST pulldowns; IB; MS. *Interaction partner*: GlyR α2 ICD. *Functional relevance*: Unknown, but GlyR‐dependent inhibition of GABA‐evoked responses is mediated by dephosphorylation of GABA_A_Rs by calcineurin. *Key reference*: Bluem et al. (2007)	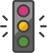
α2	MAPK1/ERK2 MAPK3/ERK1	*Role*: Mitogen‐activated protein kinases MAPK1/ERK2 and MAPK3/ERK1 are protein‐serine/threonine kinases that participate in the Ras–Raf–MEK–ERK signal transduction cascade, which regulates cell growth, adhesion, survival and differentiation. MAPK1/ERK2 and MAPK3/ERK1 were identified in GST‐GlyR α2 pulldowns from rat brain extracts using immunoblotting. *Evidence*: GST pulldowns; IB. *Interaction partner*: GlyR α2 ICD. *Functional relevance*: Unknown. No significant increase of MAPK activation upon glycine application to cultured neurons, MAPK/ERK kinase inhibitors did not influence rpS6 phosphorylation. *Key reference*: Bluem et al. (2007)	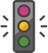
α3L	SEC8	*Role*: SEC8 is a component of the exocyst complex that mediates the tethering of post‐Golgi secretory vesicles to the plasma membrane. SEC8 was identified in GST‐GlyR α3L pulldowns from mouse brain extracts using MS. Recombinant eGFP‐tagged SEC8 co‐localized with and facilitated axonal trafficking of HA‐tagged GlyR α3L in primary hippocampal neurons. *Evidence*: GST pulldowns; IB; IC; MS; eGFP‐SEC8 fusion proteins. *Interaction partner*: GlyR α3L binds to the SEC8 via the motif ^325^TEAFALEKFYRFSDT^339^ in the TM3‐TM4 ICD. *Functional relevance*: SEC8 facilitates axonal trafficking of GlyR α3L‐SEC8 complexes in cargo vesicles destined for presynaptic glutamatergic terminals. *Key reference*: Winkelmann et al. (2014)	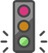
β	Gephyrin	*Role*: Gephyrin is vital for synaptic clustering of heteromeric GlyRs, selected GABA_A_R subtypes and MOCO synthesis. *Evidence*: Strychnine affinity purification, GST pulldowns, mutagenesis, yeast‐two hybrid, isothermal titration calorimetry, peptide pulldowns, immunoblotting, 3D structures. *Interaction partner*: GlyR β binds to the gephyrin E domain via the motif ^398^FSIVGSLPRDFELS^411^ in the TM3‐TM4 ICD. GlyR β‐gephyrin interactions can be modulated by PKC phosphorylation at S403. *Functional relevance*: Essential component of heteromeric GlyR complexes responsible for synaptic localization. *Key references*: Prior et al. (1992); Meyer et al. (1995); Rees et al. (2003); Harvey, Duguid et al. (2004); Schrader et al. (2004); Sola et al. (2004); Kim et al. (2006); Maric et al. (2011); Specht et al. (2011)	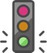
β	Neurobeachin	*Role*: Neurobeachin is a large brain specific A‐kinase anchor protein (AKAP) that targets the activity of protein kinase A to specific subcellular sites interacts with disks large MAGUK scaffold protein 3 (DLG3/SAP102) and gephyrin. Neurobeachin was identified in GST‐GlyR β pulldowns from rat brain extracts using MS. Nbea immunoreactivity colocalized with marker proteins gephyrin and GlyRs at inhibitory synapses in cultured hippocampal neurones. *Evidence*: GST pulldowns; IB; IC; MS. *Interaction partner*: direct via GlyR β ICD; indirect via gephyrin C domain. *Functional relevance*: Neurobeachin regulates receptor downscaling at inhibitory synapses in a protein kinase A‐dependent manner. *Key references*: Del Pino et al. (2011); Lutzenkirchen et al. (2024)	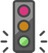
β	Vacuolar protein sorting 35 (VPS35)	*Role*: Vacuolar Protein Sorting 35 (VPS35) is a core component of the retromer cargo‐selective complex, which transports selected transmembrane cargo proteins between vesicular structures (e.g., endosomes, lysosomes, vacuoles) and the Golgi apparatus. VPS35 was identified in GST‐GlyR β pulldowns from rat brain extracts. *Evidence*: GST pulldowns; IB; MS. *Interaction partner*: direct via GlyR β ICD. *Functional relevance*: Unknown, VPS35 may be involved in GlyR internalization/recycling? *Key reference*: Del Pino et al. (2011)	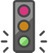
Unknown	Glypican 1 (GPC1)	*Role*: Glypicans GPC1‐GPC6 are a family of proteoglycans that are bound to the cell surface by a glycosylphosphatidyl‐inositol anchor. GPC1 was identified in IPs with GlyR α1, β and gephyrin antibodies and GlyR β BN‐PAGE from rat brainstem extracts. *Evidence*: IP; BN‐PAGE; MS. *Interaction partner*: unknown. *Functional relevance*: Unknown, but glypicans are integral components of synapse‐organizing protein complexes. *Key reference*: van der Spek et al. (2020)	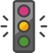
Unknown	IQSEC2	*Role*: IQSEC2 (aka BRAG1/IQ‐ArfGEF) is a neuronal ArfGEF for Arf GTPases that typically localizes to excitatory synapses as part of the NMDA receptor complex, via direct interaction with disks large MAGUK scaffold protein 4 (DLG4/PSD‐95/SAP90). IQSEC2 was identified in IPs with GlyR α1, β and gephyrin antibodies and GlyR β BN‐PAGE from rat brainstem extracts. *Evidence*: IP; MS. *Evidence*: IP; BN‐PAGE; MS. *Interaction partner*: unknown. *Functional relevance*: Unknown. *Key reference*: van der Spek et al. (2020)	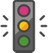
Unknown	IQSEC3	*Role*: IQSEC3 (aka BRAG3/SynArfGEF) is a neuronal ArfGEF for Arf GTPase Arf6, localizes at inhibitory synapses and interacts with gephyrin, utrophin/dystrophin and S‐SCAM/MAGI‐2. IQSEC3 was identified in IPs with GlyR α1, β and gephyrin antibodies and GlyR β BN‐PAGE from rat brainstem extracts. *Evidence*: IPs; BN‐PAGE; MS; YTH. *Interaction partner*: Likely indirect—IQSEC3 amino acids 160–210 interact with the gephyrin G domain. *Functional relevance*: IQSEC3 is vital for inhibitory synapse density, synaptic transmission and correct matching of PSDs to pre‐synaptic terminals. *Key reference*: van der Spek et al. (2020)	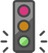
Unknown	Neurexin 3	*Role*: Neurexin 3 is primarily localized in the presynaptic membrane and interacts with LRRTMs, neuroligin‐1, neuroligin‐2, neuroligin‐3, neuroligin‐4X, CASK, cerebellin (Cbln), SHANK2 and disks large MAGUK scaffold protein 4 (DLG4/PSD‐95/SAP90). Neurexin 3 was identified in IPs with GlyR α1, β and gephyrin antibodies and GlyR β BN‐PAGE from rat brainstem extracts by mass spectroscopy. *Evidence*: IP; BN‐PAGE; MS. *Interaction partner*: Possibly indirect via GlyR β‐gephyrin‐neuroligin 2‐neurexin 3, although could be direct as neurexin 3 can bind directly to GABA_A_R ECDs. *Functional relevance*: Neurexin 3 is a key synaptic organizer at inhibitory synapses. Direct or indirect interactions with GlyRs? *Key reference*: van der Spek et al. (2020)	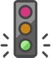

*Note:* Traffic light system indicates confidence in the interacting proteins based on available evidence: Green: Interactor with extensive supporting evidence for functional relevance; Amber: Interactor identified with a single method, with limited or unknown functional relevance; Red: Potential artifactual interactor.

Abbreviations: BN‐PAGE, blue native PAGE; GST pulldowns, GlyR‐GST fusion protein pulldowns; IB, immunoblotting; IC, immunocytochemistry; IP, immunoprecipitation; MS, mass spectroscopy; PLA, proximity ligation assay; PPI, protein–protein interaction; YTH, yeast two‐hybrid system.

## 2 Gephyrin—The Original GlyR Accessory Protein

**FIGURE 2:**
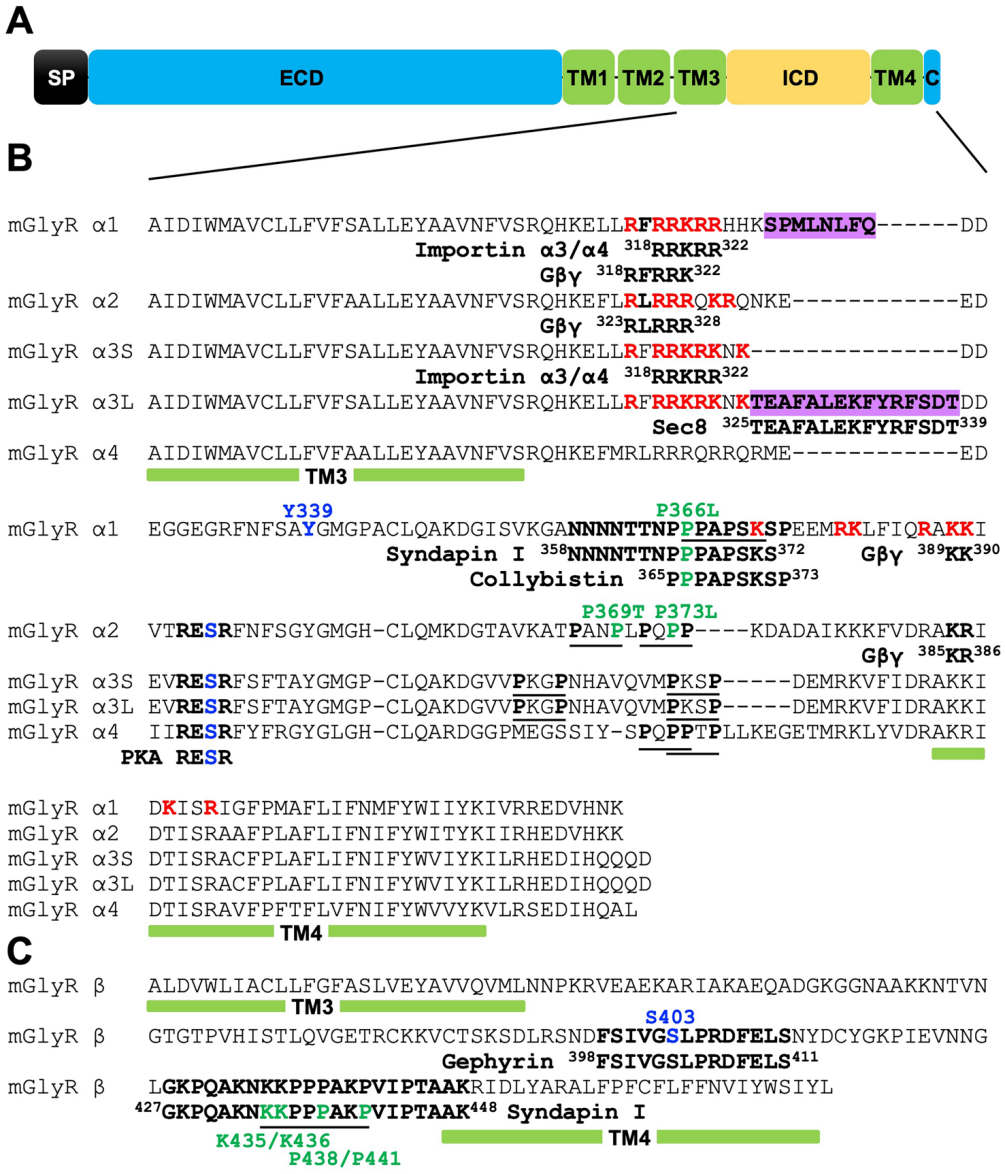
GlyR subunit functional domains and interactor binding sites. (A) GlyR subunits domain structure showing a N‐terminal signal peptide (SP), extracellular domain (ECD), membrane‐spanning domains (M1‐M4), intracellular domain (ICD) and the extracellular C‐terminus (C). Note that to date only interactors of the GlyR TM3–TM4 ICDs have been identified, which represent 17%–20% of GlyR α and 24% of GlyR β subunit polypeptides. (B) Alignment of mouse GlyR α1–α4 TM3–TM4 ICD sequences showing interactor binding sites for collybistin, importins, Gβγ, SEC8, and syndapin I (bold lettering). Note that proposed binding sites for Gβγ/importins and syndapin I/collybistin overlap in GlyR α subunits. (C) Mouse GlyR β TM3–TM4 ICD sequence depicting binding sites for gephyrin and syndapin I. For panels B and C, amino acid numbering refers to the mature sequence of mouse GlyR α1S, α2, α3S, α4 and β subunits after signal peptide cleavage. The positions of TM3 and TM4 are shown by green lines, and alternatively spliced sequences in GlyR α1L and GlyR α3L are highlighted by purple shading. Red lettering indicates charged residues, and blue lettering indicates key phosphorylation sites. Green lettering indicates GlyR α1^P366L^ implicated in startle disease and GlyR α2^P369T^ and α2^P373L^ implicated in NDDs that form part of proline‐rich motifs.

Gephyrin is perhaps best known for its role in synaptic clustering of GlyRs, but it also has a key role in the synthesis of molybdenum cofactor (Moco) required for the activity of enzymes sulfite oxidase, xanthine oxidase, and aldehyde oxidase (Feng et al. 1998; Fritschy et al. 2008). Gephyrin knockdown using antisense oligonucleotides in spinal cord neurons or in knockout mice prevents GlyR clustering (Kirsch et al. 1993; Feng et al. 1998; Fischer et al. 2000). Consistent with this role, gephyrin knockout mice die shortly after birth, exhibiting a phenotype that is mimicked by strychnine intoxication, including a rigid, hyperextended posture and apnea (Feng et al. 1998). Gephyrin forms multimers via a trimeric N‐terminal G domain, a central alternatively spliced C domain, and dimeric C‐terminal E domain (Prior et al. 1992; Rees et al. 2003; Harvey, Duguid et al. 2004; Sola et al. 2004). The GlyR β subunit binds to the gephyrin E domain via the short motif ^398^FSIVGSLPRDFELS^411^ in the TM3‐TM4 ICD (Figure 2; Kirsch et al. 1995; Meyer et al. 1995; Rees et al. 2003; Harvey, Duguid et al. 2004; Schrader et al. 2004; Sola et al. 2004; Kim et al. 2006). Mutagenesis studies revealed that hydrophobic interactions formed by F330, Y673, and P713 of gephyrin and F398 and I400 of the GlyR β ICD are critical for binding (Kim et al. 2006; Maric et al. 2011). Within this core gephyrin‐binding motif lies a protein kinase C (PKC) phosphorylation site (S403), which, when phosphorylated, reduces the affinity of binding between GlyR β and gephyrin, facilitating the lateral diffusion of GlyRs away from inhibitory postsynaptic densities (Specht et al. 2011). However, although a vital component of GlyR complexes, gephyrin is also part of the problem in GlyR proteomics. First, gephyrin is a large protein that binds to GlyR β with high affinity (Schrader et al. 2004; Kim et al. 2006; Specht et al. 2011) meaning that: (i) gephyrin peptides are over‐represented in mass spectrometry (MS) analysis of GlyR IPs or GST pulldowns and (ii) other lower‐affinity GlyR β interactors might be missed. Second, gephyrin is a *highly promiscuous protein*, interacting with microfilament components such as G‐actin, profilin, Mena/VASP (Giesemann et al. 2003), dynein light chains 1 and 2 (Fuhrmann et al. 2002), tubulin (Kirsch et al. 1991) and several other synaptic proteins including the RhoGEF collybistin (Kins et al. 2000; Grosskreutz et al. 2001; Harvey, Duguid et al. 2004), IQSEC3 (Um et al. 2016; van der Spek et al. 2020), neurobeachin (Lutzenkirchen et al. 2024), neuroligins 2 and 4 (Poulopoulos et al. 2009; Hoon et al. 2011) and importantly the TM3‐TM4 ICDs of GABA_A_R α1, α2, α3 and α5 subunits (Tretter et al. 2008, 2011; Saiepour et al. 2010; Mukherjee et al. 2011; Maric et al. 2011, 2014; Brady and Jacob 2015). The latter is consistent with a region‐specific loss of clustering of some, but not all, GABA_A_R subtypes in gephyrin‐deficient mice (Kneussel et al. 1999, 2001; Fischer et al. 2000; Lévi et al. 2004), but it means that gephyrin is a key synaptic clustering protein *at both glycinergic and GABAergic synapses*. To put this issue into a proteomic context, a recent in vivo proximity‐labeling approach for discovering inhibitory postsynaptic proteins used a modified biotin ligase enzyme (BioID)‐tagged gephyrin construct to covalently tag proteins with biotin that were within 10 nm (Uezu et al. 2016). This resulted in the identification of 91 proteins from mouse brain, including known gephyrin interactors such as collybistin, dynein light chains 1 and 2, IQSEC3, GABA_A_R α3 and neuroligin 2 (Uezu et al. 2016). However, several known gephyrin‐interacting GABA_A_R (α1, α2, α5) and the GlyR β subunit were missing in the BioID‐tagged gephyrin analysis, underscoring the importance of using receptor subtype‐specific approaches and relevant tissues/developmental stages for BioID studies.

## 3 Other GlyR β Subunit Interactors—Neurobeachin, VPS35, and Syndapin I

GlyR TM3‐TM4 ICDs also contain consensus sites for ubiquitination (Büttner et al. 2001), phosphorylation by protein kinases (e.g., PKC, PKA, tyrosine kinases; Harvey, Depner et al. 2004; Specht et al. 2011; Bai et al. 2024), and importantly for GlyR proteomics, polyproline helix type II (PPII) motifs (Langlhofer and Villmann 2016). Notably, proline‐rich motifs are present in all GlyR α and β subunits, although they are different in terms of sequence (Figure 2). These observations have led to efforts to discover additional stable and transient GlyR accessory protein interactions. Many of these approaches have focused on using GlyR TM3‐TM4 ICDs as baits in glutathione S‐transferase (GST) pulldown assays (e.g., Del Pino et al. 2011, 2014; Bluem et al. 2007). Using a GST‐GlyR β ICD bait, Del Pino et al. (2011) identified vacuolar protein sorting 35 (VPS35) and neurobeachin (NBEA) as novel interacting proteins using mass spectrometry. These interactions were subsequently confirmed using recombinantly expressed proteins (e.g., His_6_‐VPS35, EGFP‐NBEA). Interestingly, this analysis revealed that VPS35 also bound to gephyrin, while neurobeachin was proposed to exclusively interact with the GlyR β subunit (Del Pino et al. 2011). Neurobeachin is a large brain‐specific A‐kinase anchor protein (AKAP) that targets the activity of protein kinase A to specific subcellular sites. Neurobeachin contains Armadillo repeat, Beige and Chediak‐Higashi (BEACH), pleckstrin homology (PH), and WD40 repeat domains. Neurobeachin interacts with Dlg3 (SAP102), a member of the membrane‐associated guanylate kinase protein family, and is required for synaptic surface expression of AMPARs, NMDARs, and GABA_A_Rs (Nair et al. 2013; Farzana et al. 2016). It has also recently been demonstrated that neurobeachin binds directly to gephyrin, via two non‐linear motifs in the gephyrin C domain: ^176^VKEVHDELEDLPSPP^190^ and ^361^KAFITVLEMTPVLGT^375^ (Lutzenkirchen et al. 2024). *Hence, both neurobeachin and VPS35 appear to interact with both GlyR β and gephyrin*. VPS35 is a core component of the retromer cargo‐selective complex (CSC, comprising VPS26, VPS29 and sorting nexins SNX1/SNX2) that is responsible for transporting select transmembrane cargo proteins between vesicular structures (e.g., endosomes, lysosomes, vacuoles) and the Golgi apparatus (Burd and Cullen 2014). While the functional relevance of the GlyR β‐VPS35 interaction is currently unknown, VPS35 is involved in the synaptic trafficking of several other neurotransmitter receptors. For example, VPS35 was identified in a proteomic study of GABA_A_R α2‐containing proteins (Nakamura et al. 2016). In addition, VPS35 localizes to dendritic spines and influences the trafficking of excitatory AMPARs (Munsie et al. 2015; Temkin et al. 2017).

Despite these findings, it is noteworthy that neither neurobeachin nor VPS35 contain a SH3 domain. Undeterred, Del Pino et al. (2014) went on to discover a third GlyR β‐interacting protein: syndapin I. Syndapin I is a synaptically enriched protein implicated in membrane remodeling, endosomal/vesicle trafficking, and actin cytoskeletal dynamics (Quan and Robinson 2013). Importantly, syndapin I contains an N‐terminal Fes/CIP4 homology Bin‐Amphiphysin‐Rvs (F‐BAR) domain, which forms a coiled coil that mediates membrane binding and membrane tubulation, and a *C‐terminal SH3 domain*. Syndapin I co‐immunoprecipitated with native GlyRs from brainstem extracts, and the binding sites were mapped to the syndapin I SH3 domain and GlyR β ICD residues ^427^GKPQAKN **KK**PP**P**AK**P** VIPTAAK^448^. Since a KKXXPXXP motif encompassing K434/K435 and P438/P441 was evident (underlined, bold), double mutations K434A/K435A and P438A/P441A were tested for interactions with His_6_‐tagged syndapin I in GST pulldown experiments. For GST‐GlyR β^K434A/K435A^ the interaction with syndapin I was abolished, while for GlyR β^P438A/P441A^ the interaction was significantly reduced (Del Pino et al. 2014). Punctate syndapin I immunoreactivity was also demonstrated at glycinergic, GABAergic, and mixed glycinergic/GABAergic synapses in cultured embryonic rat spinal cord neurons (Del Pino et al. 2014). Moreover, acute syndapin I knockdown using viral miRNA expression resulted in a significant reduction in postsynaptic GlyR cluster size and number, an effect that could be overcome by a siRNA‐resistant myc‐tagged syndapin I construct (Del Pino et al. 2014). More recent functional assays have demonstrated that depleting syndapin I expression causes fragmentation of GlyR fields, that is, increases the dispersal and mobility of GlyR β clusters (Tröger et al. 2022). Curiously, phosphorylation of GlyR β S403, known to be triggered by synaptic signaling, caused a decoupling of GlyRs from gephyrin scaffolds that simultaneously promoted the association of GlyR β with syndapin I (Tröger et al. 2022).

Langlhofer et al. (2020) recently added another layer to the GlyR/syndapin I story by demonstrating that a PXXPXR/K motif in the GlyR α1 subunit (in the peptide ^364^NNNNTTNPPPAPSKS^372^; Figure 2) also mediates binding to syndapin I. It is noteworthy that this peptide has lower relative affinity compared to a similar peptide containing the GlyR β binding site ^433^NKKPPPAKPVIP^444^ (relative syndapin I binding α1: 0.4 versus β: 0.8; Langlhofer et al. 2020). Again, this interaction was mediated by the syndapin I SH3 domain. Moreover, a startle disease‐associated missense variant in the motif, GlyR α1^P366L^ (Figure 2) reduced the binding of GlyR α1 to syndapin I in peptide microarray assays (relative syndapin I binding to wild‐type and P366L 364–372 peptides: 0.4 versus 0.03). However, GlyR α1^P366L^ retained the ability to bind syndapin I in immunoprecipitation assays with mAb2b (Langlhofer et al. 2020). In addition, when MS was used to compare pulldowns with resin‐bound peptides for GlyR α1, GlyR α1^P366L^ and GlyR β, all fragments (including α1^P366L^) enriched syndapin I (Langlhofer et al. 2020). Label‐free quantitative MS/MS also revealed that both GlyR α1 and GlyR α1^P366L^ peptides enriched syndapin I, with no significant difference in the extent of enrichment.

Whole‐cell expression levels of GlyR α1^P366L^, α1^P366C^, α1^P366A^, α1^P366R^, and α1^P366W^ were comparable to wild‐type GlyR α1, suggesting that side‐chain volume and polarity at this position did not influence receptor trafficking or expression. However, in electrophysiological assays, GlyR α1^P366L^ caused a decrease in the I_max_ of homomeric α1 and heteromeric α1β GlyRs (to 64 ± 9 and 61% ± 8% of wild‐type levels respectively) and enhanced receptor desensitization (165% ± 18% of wild‐type values), suggesting less Cl^−^ flux in the presence of extracellular glycine. Although no differences were observed for synaptic current amplitudes, rise times, or decay times in artificial synapses, single‐channel analysis revealed a reduced unitary conductance accompanied by spontaneous channel opening events *in the absence of glycine* for both GlyR α1^P366L^ and GlyR α1^P366L^β (Langlhofer et al. 2020). Finally, in virally infected primary hippocampal cultures, GlyR α1^P366L^, but not wild‐type GlyR α1 caused accumulation of endogenous syndapin I in the cell soma (Langlhofer et al. 2020). Given the multiple effects of the GlyR α1^P366L^ mutation, and the different behavior of the GlyR α1^P366L^ mutation in peptide arrays, immunoprecipitation, and label‐free quantitative MS/MS assays, it is not possible to state with certainty that disrupted GlyR α1‐syndapin I interactions are the cause of startle disease in this case. Notably, spontaneous GlyR activity has also been identified as a potential cause of startle disease for other autosomal‐dominant inherited GlyR α1 mutations (Chung et al. 2010; Bode et al. 2013).

## 4 GlyR α1 Subunit Interactors—Collybistin, GPR39, HUWE1, Importins α3/α4 and Gβγ

### 4.1 Collybistin

The RhoGEF collybistin localizes to inhibitory synapses and contains an N‐terminal SRC homology 3 (SH3) domain, a catalytic RhoGEF domain, and a pleckstrin homology (PH) domain (Kins et al. 2000; Harvey, Duguid et al. 2004; Patrizi et al. 2012). Collybistin exists as multiple splice variants in vivo (CB1, CB2, CB3), some of which lack the SH3 domain (e.g., CB2_SH3‐_; Harvey, Duguid et al. 2004; George et al. 2021). Collybistin is responsible for delivering gephyrin to inhibitory synapses, and artificial or X‐linked intellectual disability/epilepsy mutations affecting the SH3 or PH domains cause mis‐localization of gephyrin and inhibitory receptors (Kins et al. 2000; Harvey, Duguid et al. 2004; Kalscheuer et al. 2009; Reddy‐Alla et al. 2010; Chiou et al. 2019). Collybistin knockout mice exhibit a region‐specific loss of postsynaptic gephyrin and GABA_A_ receptor clusters in the hippocampus and the basolateral amygdala. However, in several other areas, for example, neocortex, striatum, medial thalamic areas, brainstem, and spinal cord, punctate gephyrin immunoreactivities did not differ between wild‐type and knockout mice (Papadopoulos et al. 2007, 2008). This suggests that collybistin‐independent mechanisms of gephyrin clustering at synapses also exist (Papadopoulos et al. 2007). The SH3 domain regulates the gephyrin‐clustering activity of collybistin (Kins et al. 2000; Harvey, Duguid et al. 2004) via autoinhibition of PH domain phosphoinositide‐binding and gephyrin‐clustering activity (Soykan et al. 2014). This autoinhibition can be relieved by the binding of several neuronal interactors to the collybistin SH3 domain, including neuroligins 2 and 4 (Poulopoulos et al. 2009; Hoon et al. 2011), the GABA_A_R α2 subunit (Saiepour et al. 2010) or the binding of the small GTPase TC10 to the PH domain (Imam et al. 2022). The isolated collybistin PH domain binds to phosphatidylinositol 3‐phosphate (PI3P) that is associated with early/sorting endosomes (Kalscheuer et al. 2009; Papadopoulos et al. 2017). However, the binding of SH3 domain or PH domain interactors appears to induce a conformational change in collybistin, allowing a phospholipid affinity switch away from PI3P towards plasma‐membrane resident PIPs, such as phosphatidylinositol (3,4)‐bisphosphate [PI(3,4)P_2_], phosphatidylinositol 4,5‐bisphosphate [PIP_2_, PI(4,5)P_2_] and phosphatidylinositol (3,4,5)‐trisphosphate [PIP_3_, PI(3,4,5)P_3_], thereby enabling synaptic collybistin and gephyrin clustering (Soykan et al. 2014; Schäfer et al. 2020; Kilisch et al. 2020).

In this context, it is notable that the GlyR α1 subunit has also recently been reported to interact with collybistin via the PH domain (Breitinger et al. 2021). In pulldown experiments, the GST‐GlyR α1 ICD was capable of interacting with recombinant collybistin isoforms CB1_SH3+_ and CB2_SH3−_, suggesting that the interaction was not dependent on the SH3 domain (Breitinger et al. 2021). Further mutagenesis revealed that the GlyR α1‐collybistin interaction was mediated by a proline‐rich motif (^365^PPPAPSKSP^373^) in the GlyR α1 ICD and the collybistin PH domain. Interestingly, the GlyR α1 collybistin‐binding motif overlaps with the minimal syndapin I‐binding region in the GlyR α1 ICD (Figure 2) and also contains the startle disease mutation GlyR α1^P366L^ (Breitinger et al. 2021). However, on expression of recombinant GlyRs in hippocampal neurons using lentivirus delivery, no significant difference was detected between wild‐type GlyR α1 and the GlyR α1^P366L^ receptors in neurites. This may be consistent with previous studies suggesting that collybistin is not required for GlyR synaptic localization (Papadopoulos et al. 2007). However, additional biochemical experiments did not clearly demonstrate that GlyR α1^P366L^ interferes with collybistin binding in vitro (Breitinger et al. 2021). Relative signals in co‐immunoprecipitation experiments of GlyR α1/collybistin versus GlyR α1^P366L^/collybistin with mAb2b were 0.6 for GlyR α1^P366L^ versus 1.1 for wild‐type GlyR α1 (*n* = 4, not significant). Hence, the functional relevance of the GlyR α1‐collybistin interaction is unclear. It is plausible that GlyR subunits could also act as collybistin activators during synaptogenesis, but whether proline‐rich motifs in other GlyR subunits (i.e., α2, α3, α4) are also capable of binding to collybistin SH3, RhoGEF, or PH domains remains to be determined. Certainly, GABA_A_R α2 is a subunit known to bind to the collybistin SH3 domain via a non‐PXXP motif (Saiepour et al. 2010; Hines et al. 2018). This interaction is critical for the targeting of α2‐containing GABA_A_Rs to the axon initial segment, and disrupting GABA_A_R α2‐collybistin interactions results in increased susceptibility to seizures, early mortality, deficits in working and recognition memory, hyperactivity, anxiety, and reduced social preference (Hines et al. 2018, 2022).

### 4.2 GPR39

G protein‐coupled receptor 39 (GPR39) is an orphan G‐protein coupled receptor that signals via Gq, Gs, G12/13, Gi, and β‐arrestin to induce signaling pathways that regulate cellular survival, proliferation, differentiation, and ion transport (Doboszewska et al. 2024). GPR39 has constitutive activity and Zn^2+^ and/or eicosanoids (15‐HETE and 14,15‐EET) are candidate ligands (Doboszewska et al. 2024). GPR39‐GlyR α1 interactions were investigated by Bai et al. (2024), who discovered that GPR39 was integral to normal glycinergic input onto spinal cord somatostatin‐positive (SOM+) interneurons that are critical for the processing of mechanical pain stimuli. GPR39‐GlyR α1 interactions were first detected using a proximity ligation assay (PLA), which allows detection of protein–protein interactions in situ (at distances < 40 nm; Bai et al. 2024). PLA exploits specific antibodies identifying two proteins of interest with covalently linked specific DNA primers. A hybridization step followed by a PCR amplification with fluorescent probes then permits visualization of spots of proximity by fluorescence microscopy. GPR39‐GlyR α1 PLA revealed dense punctate signals in somatostatin‐positive interneurons that were absent if the GlyR α1 antibody was omitted. In GST‐pulldown experiments, the GST‐GlyR α1 ICD, but not the GST‐GlyR α3 or GST‐GlyR β ICDs, purified FLAG‐tagged GPR39 overexpressed in non‐neuronal cells (Bai et al. 2024). The GlyR α1 binding site was localized to the intracellular C‐terminus of GPR39. Viral expression of the GPR39 C‐terminus in spinal cord somatostatin‐positive interneurons enhanced the amplitudes of GlyR‐mIPSCs, while targeted knockdown of GPR39 led to GlyR α1 tyrosine dephosphorylation at Y339 (Figure 2) and reduced glycinergic inhibition (Bai et al. 2024). This suggests a role for GPR39 modulation of GlyR α1‐containing receptors in the stabilization of normal glycinergic transmission in pathways vital for processing mechanosensory information. In this context, it is notable that GlyR α3 activity is modulated by different GPCRs (prostaglandin EP2R and 5HT1AR) linked to protein‐kinase A signaling pathways, modulating inflammatory pain sensitization and rhythmic breathing (Harvey, Depner et al. 2004; Manzke et al. 2010). However, in contrast to GPR39‐GlyR α1, there is no evidence to date that either of these GPCRs directly interact with GlyR α3.

### 4.3 HUWE1

Ubiquitination is an important mechanism for turnover of synaptic receptors, and an elegant study by Büttner et al. (2001) demonstrated that GlyR ubiquitination of lysine residues in the TM3‐TM4 ICD precedes internalization and proteolytic cleavage of plasma membrane‐bound GlyRs. However, the identity of the E3 ubiquitin‐protein ligase that mediates ubiquitination and subsequent proteasomal degradation of GlyRs was unknown. However, Zhang et al. (2019) demonstrated a direct interaction of GlyR α1 with HUWE1 (HECT, UBA and WWE domain containing E3 ubiquitin protein ligase 1). GlyR α1, but not GlyR α3, was detected using a HUWE1 antibody in immunoprecipitation experiments using mouse spinal cord lysates. In reciprocal experiments, HUWE1 could be co‐immunoprecipitated by a GlyR α1 antibody (Zhang et al. 2019). In non‐neuronal cells, co‐expression of recombinant HUWE1 and ubiquitin enhanced the ubiquitination of GlyR α1, but not GlyR α3. HUWE1 co‐localized with gephyrin at inhibitory synapses, and HUWE1 knockdown via shRNA prevented NMDAR‐mediated increases in GlyR α1 ubiquitination in mouse neurons. Amplitudes of GlyR mIPSCs were larger in shHUWE1‐expressing neurons relative to those expressing control shRNAs (Zhang et al. 2019). Taken together, these results suggest that HUWE1 is the E3 ubiquitin ligase responsible for regulating synaptic density of α1β GlyRs, but that other GlyR subtypes (e.g., α3β) may be regulated by different mechanisms.

### 4.4 Importins α3/α4 and G Protein βγ

Importin α and β subunits mediate nuclear import of proteins containing classical nuclear localization signals (NLS; Melzer et al. 2010). Yeast two‐hybrid assays and GST pulldown experiments detected interactions of importins 3 and 4 with GAL4BD‐ICD or GST‐ICD fusion proteins for GlyR α1, α3S, α3L, but not GlyR α2 or β subunits. The importin binding site was localized to a nuclear localization signal‐like motifs present in GlyR α1 (^318^RRKRR^322^) and α3 (^318^RRKRK^322^) ICDs (Figure 2), which differ in GlyR α2 (RRRQK) and α4 (RRRQR) subunits (Melzer et al. 2010). Interestingly, eGFP‐GlyR α1 and eGFP‐α3 ICD fusion proteins show nuclear sorting in non‐neuronal and neuronal cells, which can be eliminated on mutagenesis of the NLS. However, no clear association of GlyR α1 was observed with importins 3 and 4 in immunocytochemistry of spinal cord neuronal cultures (Melzer et al. 2010) and GlyR‐importin interactions have not been observed in GlyR immunoprecipitation studies (van der Spek et al. 2020). Hence, GlyR‐importin interactions may be artifactual as isolated ICDs may show false‐positive interactions in the yeast two‐hybrid system (Bruckner et al. 2009) or mis‐localization to cellular compartments that are not representative of the behavior of full‐length GlyR subunits (Melzer et al. 2010). However, it is important to note that the NMDAR GluN1 harbors a cytoplasmic NLS that tethers importin α subunits to PSDs in a phospho‐dependent manner (Grochowska et al. 2021), providing a mechanism for the nuclear import of cargos important for transcription‐dependent forms of neuronal plasticity.

However, at least two other biological roles have been postulated for this stretch of charged amino acids in the GlyR α1 ICD. First, ICD residues ^316^RFRRK^320^ and ^385^KK^386^ are known to be critical for binding cytosolic G‐protein βγ subunits, which in turn enhance currents mediated by recombinant GlyR α1 and functional allosteric modulation by ethanol at behaviorally relevant concentrations (Yevenes et al. 2006, 2008, 2010). A peptide containing the ^316^RFRRKRR^322^ motif was able to inhibit binding of Gβγ to the GlyR α1 ICD and decreased positive modulation by ethanol (Yevenes et al. 2008). GlyR α2 also binds Gβγ via similar motifs (ICD residues ^316^RFRRK^320^ and ^385^KR^390^) but requires co‐expression of GlyR β for effective Gβγ and ethanol modulation (Muñoz et al. 2021). Knock‐in mice containing mutations GlyR α1^K385A/K386A^ or GlyR α2^K389A/R390A^ that disrupt G‐protein βγ interactions (Yevenes et al. 2010) had reduced ethanol sensitivity, faster recovery from a sedative dose of ethanol, and higher ethanol intake in the drinking in the dark protocol (Aguayo et al. 2014; Gallegos et al. 2021). Second, the charged residues ^316^RFRRKRR^322^ are located within a proposed MX‐helix (GlyR α1S H311‐G329) at the start of the GlyR α1 ICD that governs membrane topology and influences receptor function (Sadtler et al. 2003). Evidence for this has recently been provided by a cryo‐EM study of the GlyR α1β (Gibbs et al. 2023) which suggested that GlyR ICDs form a post‐M3 juxta‐membrane MX α‐helix that runs parallel to the cytosolic membrane interface, and a long extended pre‐M4 MA helix, which descends from the membrane with each subunit joining a helix bundle along the pore axis. Hence, these positively charged residues may electrostatically attract incoming anions to the intracellular compartment (Gibbs et al. 2023). This theory is also supported by a study in which eight positively charged residues in the pre‐TM4 MA helix were subjected to charge reversal by mutation to negatively charged glutamic acid, causing either a total loss of function (all eight) or modest decreases in GlyR conductance (92.2 ± 2.8 to 60.0 ± 2.2 picosiemens) for the quadruple substitution R377E/K378E/K385E/K386E (Carland et al. 2009). Taken together, these studies suggest that charged residues in the MX and MA helices control GlyR topology and form portals that contribute to the GlyR Cl^−^ permeation pathway. This is further secondary evidence that the GlyR α1/α3‐importin interaction is potentially artifactual.

## 5 GlyR α2 Subunit Interactors

In order to identify interactors of the embryonic GlyR isoform, a GST‐GlyR α2 ICD fusion protein was used as bait in pulldown assays using rat brain extracts (Bluem et al. 2007). Interacting proteins were subsequently analyzed by mass spectrometric analysis and immunoblotting (Bluem et al. 2007). This approach yielded an eclectic assortment of proposed interactors including proteins associated with translational machinery including eukaryotic elongation factor 1 α2 (eEF1α2), p70 ribosomal S6 protein kinase and ribosomal S6 protein (rpS6), as well as mitogen‐activated protein kinase extracellular signal‐regulated kinase (MAPK ERK1/2) and calcineurin (Bluem et al. 2007; Table 1). Despite earlier successes with GlyR β interactors, it is notable that identifying PPIs using a GST‐fusion protein pulldown approach often produces false positives that do not represent physiologically relevant PPIs (Wissmueller et al. 2011). Additionally, true direct interactors can fail to be affinity purified due to factors such as improper folding or lack of post‐translational modification of bait proteins (Mackay et al. 2007). Indeed, mass spectrometric analysis of the interactome captured by the GST‐GlyR α2 ICD discovered several other proteins, including LANCL1, eEF1Bγ, CRMP4, Nedd4 and nardilysin, which further analysis revealed to be non‐specific interactors of GST (Bluem et al. 2007). Follow‐up from this study has been difficult, as many of the primary proteins were not unequivocally identified. This highlights the need to publish primary data in GlyR proteomic studies. For example, calcineurin is a Ca^2+^ and calmodulin‐dependent serine/threonine protein phosphatase and is a heterodimer of a calmodulin‐binding catalytic subunit, calcineurin A (61 kDa) and a Ca^2+^‐binding regulatory subunit, calcineurin B (19 kDa). The calcineurin isoform identified by Bluem et al. (2007) had a molecular weight of 58 kDa, but there are three isozymes of the calcineurin A subunit (PPP3CA, PPP3CB, PPP3CC), and the specific isoform interacting with GlyR α2 was not identified. For the other interactors, GlyR activation in spinal cord cultures resulted in an increased phosphorylation of rpS6 and partial overlap of rpS6 and eEF1α2 with synaptic GlyRs. However, follow‐up analyses employing purified eEF1α2 and GST‐GlyR α2 failed to demonstrate a direct interaction (Bluem et al. 2007) and no subsequent study has studied the functional relevance of these interactions using different proteomic or functional techniques. In our view, it is timely to revisit the proteomics of GlyR α2, as disrupted GlyR α2‐accessory protein PPIs may have relevance to neurodevelopmental disorders. Marcogliese et al. (2022) recently reported two NDD probands with ASD, DD, ID and epilepsy who carry missense variants p.P369T or p.P373L in the hemizygous state in males. Notably, these GlyR α2 variants form part of a tandem PXXP motif within the GlyR α2 ICD (Figure 2) and are predicted to disrupt interactions with as yet uncharacterized interactors containing an SH3 domain. Thus, the identification of novel GlyR α2 interactors may lead to advances in the understanding of intracellular signaling pathways relevant to human disease.

## 6 GlyR α3 Interactors—SEC8, a Component of the Exocyst Complex

With the exception of the importins (Melzer et al. 2010) few studies have investigated interactors of the GlyR α3 subunit, which is surprising given the key roles of this subtype in central inflammatory pain sensitization and rhythmic breathing. GlyR α3 is known to be modulated indirectly by GPCRs, including the prostaglandin EP2 and 5‐HTA_1A_ receptor signaling pathways that affect protein kinase A‐mediated phosphorylation of GlyR α3^S346^ within the ICD motif RE**S**R (Harvey, Depner et al. 2004; Manzke et al. 2010; Figure 2). However, GlyR α3 also exists in two splice variants, here denoted as GlyR α3S and α3L, that differ by the absence or presence of a 15 amino acid motif ^325^TEAFALEKFYRFSDT^339^ in the TM3‐TM4 ICD (Nikolic et al. 1998; Figure 2). In cell lines, GlyR α3S exhibits a diffuse membrane localization and fast desensitization kinetics, while α3L exhibits a clustered membrane appearance and slow desensitization kinetics (Nikolic et al. 1998; Notelaers et al. 2012, 2014). GlyR α3S/α3L splice variant ratios are altered in temporal lobe epilepsy (TLE), with up‐regulation of α3S relative to α3L in TLE patients with a severe course of disease and a high degree of hippocampal damage (Eichler et al. 2009). In primary hippocampal neurons, recombinant HA‐tagged GlyR α3L clusters were more likely to co‐localize with glutamatergic (vesicular glutamate transporter VGluT‐positive) nerve endings rather than at GABAergic synapses (GAD65‐positive), while α3S was mainly in a diffuse state. A GlyR α3L‐specific antibody revealed that GlyR α3L clusters at glutamatergic nerve endings and co‐localized with VGluT (Eichler et al. 2009). Interestingly, Winkelmann et al. (2014) demonstrated the molecular basis for this differential targeting, since α3L, but not α3S, associates with SEC8, a member of the exocyst protein family of vesicular trafficking factors. A GST‐tagged α3L ICD was used to purify and identify GlyR α3L interactors from adult mouse brain using mass spectrometry. Interestingly, recombinant SEC8‐EGFP co‐localized with and facilitated axonal trafficking of HA‐tagged GlyR α3L in primary hippocampal neurons (Winkelmann et al. 2014). These results suggest that the role of the GlyR α3L‐SEC8 interaction is to facilitate axonal trafficking of GlyR α3L‐SEC8 complexes in axonal cargo vesicles targeted to presynaptic glutamatergic terminals. Lastly, it is notable that GlyR α3S, α3L and α4 subunit ICDs also contain PXXP motifs (Figure 2), but no SH3 domain‐containing interactors have been reported, with no interactors whatsoever reported for GlyR α4.

## 7 Other GlyR Interactors Identified by Immunoprecipitation—Direct or Indirect Interactors?

One major issue with immunoprecipitation studies is that GlyR interactors have been identified that may, or may not, interact directly with the target GlyR subunits. For example, in a recent study using IPs with GlyR α1, β and gephyrin antibodies from rat brainstem that also utilized GlyR β BN‐PAGE, several interactors were found where the binding partner could not readily be determined, including glypican 1, neurexin 3, and the ArfGEFs IQSEC2 and IQSEC3 (van der Spek et al. 2020). Glypicans GPC1‐GPC6 are a family of proteoglycans that are bound to the cell surface by a glycosylphosphatidyl‐inositol anchor. They are integral components of synapse‐organizing protein complexes (Kamimura and Maeda 2021) and serve as ligands for leucine‐rich repeat transmembrane neuronal proteins (LRRTMs) and leukocyte common antigen‐related (LAR) family receptor protein tyrosine phosphatases (RPTPs). For example, glypicans 4 and 6 act as astrocyte‐secreted signals to promote the formation of excitatory synapses by increasing the surface level and clustering of GluA1‐containing AMPA receptors (Allen et al. 2012). However, the binding partners of GPC1 at inhibitory glycinergic synapses are unknown. Neurexin 3 is also a synaptic organizer primarily localized in the presynaptic membrane and interacts with LRRTMs, neuroligin‐1, neuroligin‐2, neuroligin‐3, neuroligin‐4X, CASK, cerebellin (Cbln), SHANK2, and disks large MAGUK scaffold protein 4 (DLG4/PSD‐95/SAP90; Zhang et al. 2022). Neurexins are clearly involved in the maintenance of glycinergic synapses, since triple neurexin 1/2/3 conditional knockout mice reduced the amplitude and altered the kinetics of glycinergic synaptic transmission in the brainstem (Jiang et al. 2024). In this case, the assumption is that neurexin 3 interacts indirectly with GlyRs via a GlyR β‐gephyrin‐neuroligin 2‐neurexin 3 pathway (Poulopoulos et al. 2009). However, it is noteworthy that the ECDs of neurexins can bind directly to the GABA_A_R α1 subunit ECD in a Ca^2+^ dependent manner (Zhang et al. 2010). Hence, a direct GlyR‐neurexin 3 interaction cannot be excluded.

### 7.1 ArfGEFs IQSEC2 And IQSEC3

Two other synaptic proteins identified in GlyR α1, β and gephyrin and GlyR β BN‐PAGE were the ArfGEFs IQSEC2 and IQSEC3 (van der Spek et al. 2020). Both proteins have a similar structure, encompassing a calmodulin‐binding IQ‐like motif, a central Sec7 domain, a PH domain, and a C‐terminal type I PDZ‐binding motif. IQSEC2 (also known as BRAG1/IQ‐ArfGEF) is a neuronal ArfGEF for Arf GTPases that typically localizes to excitatory synapses as part of the NMDAR complex, via direct interaction with disks large MAGUK scaffold protein 4 (DLG4/PSD‐95/SAP90; Murphy et al. 2006; Sakagami et al. 2008). IQSEC2 is involved in dendritic spine morphogenesis (Hinze et al. 2017) and knockout mice have revealed defects in AMPAR‐, NMDAR‐, and GABA_A_R‐mediated synaptic transmission (Mehta et al. 2021) suggesting that IQSEC2 is also important for the function of inhibitory synapses. By contrast, IQSEC3 (also known as BRAG3/SynArfGEF) colocalizes with GlyRs and GABA_A_Rs in the retina and brain (Sakagami et al. 2013), interacts with the gephyrin G domain (van der Spek et al. 2020), utrophin/dystrophin, and S‐SCAM/MAGI‐2 (Fukaya et al. 2011). Further studies have demonstrated that IQSEC3 is vital for inhibitory synapse density, synaptic transmission, and correct matching of PSDs to pre‐synaptic terminals (Um et al. 2016; Früh et al. 2018; Kim et al. 2022). Curiously, ribosomal p70‐S6K1‐mediated signaling is up‐regulated in the hippocampus of *Iqsec3* knockout mice, providing a potential link to two other potential GlyR interactors, p70‐S6K1 and rpS6 (Bluem et al. 2007; Kim et al. 2022). IQSEC2 and IQSEC3 were also identified in two large‐scale studies of inhibitory synapse proteomics using proximity‐labeling approaches targeting gephyrin and collybistin (Uezu et al. 2016) and Designed Ankyrin Repeat Proteins (DARPins), genetically engineered antibody mimetics designed to exhibit high‐affinity binding for gephyrin (Campbell et al. 2022). This further evidence confirms the association of both IQSEC2 and IQSEC3 with inhibitory synapses. Although GlyRs are likely to associate with IQSEC3 *indirectly* via gephyrin (van der Spek et al. 2020), the role of IQSEC2 at inhibitory synapses remains unclear.

## 8 Limitations and Experimental Bias in Current GlyR Proteomics Studies

While proteomics studies to date have identified several important GlyR interactors (Figure 1; Table 1) there are clear flaws and biases in the experimental approaches that have been used to date. The majority of studies have focused on the adult GlyR α1β isoform, with few interactors identified for the GlyR α2 and α3 subunits, despite the presence of potential interaction motifs (Figure 2; Table 1). There are also no known interactors for the GlyR α4 subunit. This is not related to the relative abundance of the different GlyR subtypes, but rather a lack of experimental focus and/or availability of appropriate tools, such as GlyR subtype‐selective antisera. Many GlyR interactors have been identified only once, using a single method or using methods that are prone to false‐positive interactions, for example, yeast two‐hybrid and GST pulldowns (Bruckner et al. 2009; Wissmueller et al. 2011), with little in the way of functional follow‐up. GlyR interactors that fall into this category are calcineurin, eEF1α2, importins, p70S6K, MAPK1/3, and rpS6 (Figure 1; Table 1). Most approaches to the identification of PPIs have focused exclusively on the TM3‐TM4 ICDs, meaning that important interactors associating with GlyR ECDs, TM domains, or at GlyR αβ subunit interfaces would be missed. For example, different inhibitory GABA_A_R subtypes interact with a variety of transmembrane auxiliary subunits that either regulate receptor trafficking or modulate functional and pharmacological properties (Han et al. 2021). These auxiliary proteins include the lipoma HMGIC fusion partner‐like 3 and 4 (LHFPL3/LHFPL4; Davenport et al. 2017; Yamasaki et al. 2017), Clptm1—cleft lip and palate transmembrane protein 1 (Ge et al. 2018), Shisa7 (Han et al. 2019) and TMEM132B (Wang et al. 2024). These proteins play diverse roles, including: (i) GABA_A_R synapse‐specific targeting and clustering (LHFPL3/LHFPL4; Davenport et al. 2017; Yamasaki et al. 2017); (ii) limiting GABA_A_R forward trafficking (Clptm1, Ge et al. 2018); (iii) accelerating GABA_A_R deactivation (Shisa7; Han et al. 2019) or (iv) promoting cell‐surface expression while also slowing GABA_A_R deactivation (TMEM132B; Wang et al. 2024). However, to date, no equivalent transmembrane auxiliary subunits have been identified for GlyRs.

Although reciprocal binding sites have been identified for several GlyR interactors, these experiments are low‐throughput and time‐consuming, often involving trial‐and‐error mutagenesis of expression constructs for GlyR subunits and target interactors (Figure 2; Table 1). It is also evident that some synaptic proteins, such as collybistin, gephyrin, neuroligins, and neurobeachin, have multiple binding partners. As the sensitivity of mass spectroscopy improves, we are also likely to detect secondary or tertiary interactors (e.g., neurexin 3, IQSEC2, IQSEC3; van der Spek et al. 2020). We therefore need to integrate methods that provide rapid, high‐throughput identification of reciprocal binding sites within GlyR complexes into future studies. Consideration needs to be given to adequate controls for GST pulldown or immunoprecipitation approaches. Finally, there is a major assumption in the field that GlyRs segregate into simple homomeric or heteromeric combinations in vivo (e.g., GlyR α2, GlyR α1β, GlyR α2β, GlyR α3β, etc.). However, given that native heteromeric GlyR combinations are now thought to contain multiple GlyR α subunits in a 4α:1β stoichiometry (Yu et al. 2021; Zhu and Gouaux 2021), it is highly likely that triheteromeric GlyRs exist in vivo. In support of this theory, van der Spek et al. (2020) noted that GlyR α2 and α3 subunits co‐immunoprecipitated with GlyR α1 antisera, even though these reagents recognized GlyR α1‐specific epitopes. While it is possible that this result represents indirect immunoprecipitation of separate GlyR α1β, GlyR α2β and GlyR α3β receptors via the gephyrin lattice, a simpler explanation is that triheteromeric GlyRs (i.e., GlyR α1α2β, GlyR α1α3β) exist in vivo. This possibility needs to be taken into account in experimental design.

## 9 A Road Map for Unlocking the GlyR Interactome

**FIGURE 3:**
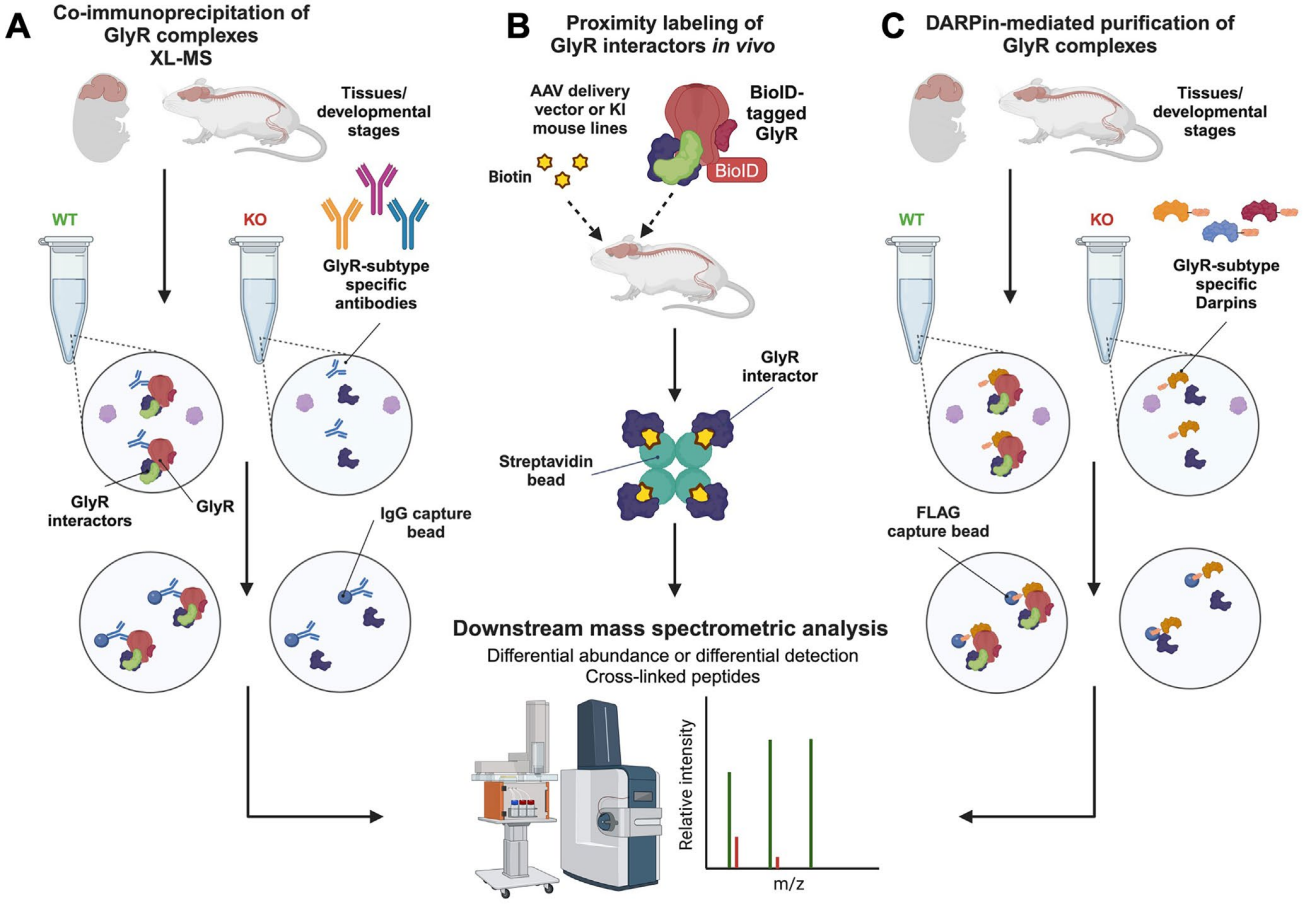
A roadmap for experimental approaches for unlocking the GlyR interactome. Novel GlyR interacting proteins can be identified using a range of modern methods coupled with downstream mass‐spectrometric analysis. (A) Native GlyR complex co‐immunoprecipitations using multiple subtype‐selective antibodies in wild‐type versus knockout mice; (B) Proximity labeling in vivo with BioID‐tagged GlyR subunits; (C) GlyR subtype‐selective Darpins recognizing different epitopes. Binding sites can then be mapped using array‐based peptide binding before proceeding to detailed follow‐up assays to validate, map, and characterize interactor relationships to GlyRs and glycinergic signaling using biochemistry, cryo‐EM, electrophysiology, shRNA‐mediated knockdown, high‐resolution microscopy, or interactor mouse knockout lines. Figure created using elements from https://BioRender.com.

Taking these limitations into account, we propose a road map for high‐throughput experimental methodologies that can be employed to reliably capture in vivo GlyR complexes (Figure 3). Novel GlyR interacting proteins can be identified using a range of modern methods coupled with downstream mass‐spectrometric analysis. These approaches could include: (1) Native GlyR complex co‐immunoprecipitations using multiple subtype‐selective antibodies in wild‐type versus knockout mice; (2) Proximity labeling in vivo with spatially restricted, proximity‐dependent biotinylation (BioID)‐tagged GlyR subunits; (3) GlyR subtype‐selective Designed Ankyrin Repeat Proteins (DARPins) recognizing different epitopes; (4) Reciprocal binding sites can then be mapped using array‐based peptide binding before proceeding to detailed functional assays.

### 9.1 Immunoprecipitation of Native GlyR α1‐α4 Complexes With Appropriate Controls

High‐throughput synaptic interaction proteomics has burgeoned since the beginning of the century, largely driven by advancements in the accuracy and sensitivity of mass spectroscopy downstream of the affinity purification of solubilized receptor complexes from native tissue (Li et al. 2010). This approach has been employed with great success in the characterization of AMPAR and GABA_A_R interactomes (Schwenk et al. 2009, 2012; Heller et al. 2012; Chen et al. 2014; Chen, Koopmans, Paliukhovich, et al. 2023; Chen et al. 2023; Nakamura et al. 2016; van der Spek et al. 2022). These investigations have identified a plethora of important interactors including transmembrane AMPAR regulatory proteins (TARPs), cornichons, Shisa family proteins, germ‐cell specific gene 1‐like protein (GSG1L), as well as SynDIG1 and SynDIG4 and GABA_A_R transmembrane proteins. These proteins can influence receptor trafficking, localization at synaptic sites, subtly modulate biophysical properties of receptors (e.g., increasing conductance, slowing down kinetics), and regulate synaptic numbers, strength, and plasticity. The methodological strengths of these high‐throughput affinity purifications have been the incorporation of robust controls, including: (i) The use of multiple target‐specific antibodies for immunoprecipitation that recognize distinct epitopes; (ii) the use of knockout tissue as a negative control; or (iii) Using gene‐editing technologies to introduce epitopes (e.g., myc, pHluorin or Venus tags) into the gene/protein of interest (Schwenk et al. 2009, 2012; Heller et al. 2012; Chen et al. 2014; Nakamura et al. 2016; van der Spek et al. 2022). For this reason, we recommend that future GlyR immunoprecipitation experiments utilize multiple target‐specific antibodies recognizing different antigens, and integrate control tissue from mouse mutant or knockout lines that lack given GlyR subunits, e.g., GlyR α1 *oscillator*, GlyR α2, GlyR α3 and GlyR α4 knockout mice (Buckwalter et al. 1994; Harvey, Depner et al. 2004; Avila et al. 2013; Nishizono et al. 2020). This will allow cross‐comparison of different result sets—with an ideal interactor immunoprecipitating with multiple antibodies from wild‐type but not knockout tissue, where the target GlyR isoform is absent. Any interactors that are immunoprecipitated from knockout tissues are likely to be non‐specific in nature. A future focus on the GlyR α2, α3 and α4 subunits is also warranted, particularly as these subtypes all contain unique proline‐rich motifs (Langlhofer and Villmann 2016) that could serve as sites for PPIs with SH3‐domain containing proteins. The identification of GlyR α2 interactors that are disrupted by the GlyR α2^P369T^ or GlyR α2^P373L^ variants (Marcogliese et al. 2022) is also of high priority in terms of understanding pathogenic mechanisms in human disease.

### 9.2 Alternative Approaches—BioID And Darpins

Spatially‐restricted, proximity‐dependent biotinylation (BioID) represents a chemico‐genetic approach to the study of interaction proteomics. The BioID approach first utilized an *E. coli* biotin ligase mutant (BirA*), which generates reactive biotin (biotinoyl‐5‐AMP) and has an enhanced off‐rate, meaning that biotin covalently attaches to exposed lysine residues of any neighboring protein within 10 nm (Roux et al. 2012; Guo et al. 2023). More recently, TurboID (35 kDa) and miniTurbo (28 kDa) were developed, which display accelerated rates of protein biotinylation compared to BioID (10 min vs. 18 h; Kim et al. 2016; Branon et al. 2018). These biotin ligases are fused to a protein of interest and expressed in cells/tissues of interest to a protein of interest in vivo or in cultured cells followed by the addition of biotin, which is covalently bound to proteins within nanometer proximity of the catalytic ligase (Takano and Soderling 2021). The biotinylated proteins are then purified by affinity isolation using streptavidin‐coupled beads and analyzed by mass spectrometry to uncover PPIs with high spatial resolution. Proximity labeling offers several benefits over the traditional co‐immunoprecipitation proteomic approach, namely capturing low‐affinity and transient interactors that are routinely lost during affinity purification (Takano and Soderling 2021). Conversely, there is a risk that the fusion of the biotin ligase to the protein of interest may alter protein trafficking or occlude PPI sites (Elhabashy et al. 2022). Moreover, because proximity labeling relies on *vicinity* rather than *location*, false positives may be generated. For example, it would be difficult to exclusively label synaptic proteins as opposed to those involved in protein trafficking. Proximity labeling has already been employed in the study of synaptic protein complexes. For example, Uezu et al. (2016) fused BioID to gephyrin, collybistin, and PSD‐95 in order to investigate the proteomes of inhibitory and excitatory postsynaptic complexes. Following injection of these fusion protein constructs encased in an adeno‐associated virus (AAV) vector and exogenous biotin supplementation, biotinylated proteins were affinity‐purified by streptavidin and identified by MS (Uezu et al. 2016). The BioID‐gephyrin fusion protein was validated as having co‐localized at inhibitory synaptic densities and biotinylated known gephyrin interactors collybistin and IQSEC3, as well as novel interactors inhibitory synaptic proteins 1 and 2 (InSyn1 and InSyn2; Uezu et al. 2016). Although known gephyrin interactors such as collybistin, dynein light chains 1 and 2, IQSEC3, GABA_A_R α3 and neuroligin 2 were identified using this method, several known gephyrin‐interacting GABA_A_R (α1, α2, α5) and the GlyR β subunit were missing (Uezu et al. 2016). A different but overlapping set of interactors was identified using BioID‐collybistin, including the gephyrin‐interacting GABA_A_R α1, α2, α3, α5 subunits (Uezu et al. 2016), suggesting that BioID analysis may be prone to *spatial bias* if interactors are not within 10 nm of the BioID tag. Certainly, for BioID‐gephyrin the tag was attached to the gephyrin G domain (Uezu et al. 2016), while GABA_A_R and GlyRs interact with the E domain. By contrast, the collybistin binding motif on gephyrin is located at the gephyrin C domain/E domain junction (Harvey, Duguid et al. 2004), so BioID‐collybistin would be closer to GABA_A_R and GlyR subunits. One other challenge in using BioID tools for GlyR proteomics will be introducing these relatively large tags into the extracellular N‐termini or TM3‐TM4 ICDs of GlyR subunits. The position of the tag would introduce a bias in favor of extracellular versus intracellular interactors, and control experiments would need to be conducted to ensure that the BioID‐tagged GlyRs show correct trafficking, synaptic localization, and function.

Designed Ankyrin Repeat Proteins (DARPins) are genetically engineered antibody mimetics that represent an interesting new tool for receptor proteomics, since they exhibit specific and high‐affinity target protein binding (Binz et al. 2003). DARPins utilize structural motifs from ankyrin repeat proteins, which contain multiple repeats of 33 amino acid modules, consisting of a β‐turn followed by two anti‐parallel α‐helices. DARPins are small (12–15 kDa) and have two or three repeat modules sandwiched between a hydrophilic N‐ and C‐terminal cap that forms a continuous hydrophobic core and a large groove‐like solvent‐accessible binding surface. Campbell et al. (2022) recently demonstrated the utility of this system for inhibitory synaptic proteomics, generating several DARPins recognizing gephyrin in phosphorylated (S268, S270) and dephosphorylated states. Combined coverage between experiments using each DARPin was used to create a common gephyrin interaction network, which was compared to interactors precipitated by the anti‐gephyrin antibody 3B11 to compile a high‐confidence consensus gephyrin interactome (Campbell et al. 2022). This network encompassed the majority of canonical gephyrin‐associated proteins, including GABA_A_ and glycine receptors, inhibitory synaptic scaffolding and adhesion molecules (e.g., collybistin, InSyn1, IQSEC2, IQSEC3, neuroligin 2, neuroligin 3), and cytoskeletal adaptor proteins (e.g., dynein light chains, Mena, VASP). However, DARPin analysis of gephyrin interactors also identified proteins involved in mRNA regulation, cytoskeletal proteins and adaptors, metabolic enzymes, and ribosomal subunits, suggesting novel functions of gephyrin beyond synaptic scaffolding and molybdenum cofactor biosynthesis (Campbell et al. 2022). The advantage of this approach for future GlyR proteomics studies is that multiple DARPins that recognize a given epitope could be generated using a synthetic combinatorial library and in vitro selection methods. These DARPins could be developed as tools to visualize GlyR subtypes at synapses in living neurons by fusing DARPins to genetically‐encoded fluorescent proteins. Indeed, Campbell et al. (2022) demonstrated the versatility of the gephyrin DARPins for synaptic biology, showing that FLAG‐tagged anti‐gephyrin DARPins correctly recognized inhibitory synapses labeled using anti‐gephyrin antibodies such as mAb7a in neuronal cultures and mouse brain tissue.

### 9.3 Mapping of Binding Sites—Cross‐Linking Mass Spectrometry (XL‐MS) and Peptide Arrays

We have highlighted the need for methods that provide rapid, high‐throughput identification of reciprocal binding sites within GlyR complexes. One possibility in this regard is the use of cross‐linking MS (XL‐MS; Liu and Heck 2015; O'Reilly and Rappsilber 2018), a method that involves the use of a soluble, MS‐cleavable cross‐linker such as disuccinimidyl sulfoxide (DSSO), followed by the downstream analysis of cross‐linked peptides via MS and a data analysis pipeline (e.g., XlinkX v2.0; Liu et al. 2017). DSSO cross‐links lysine residues in peptides separated by 10‐27 Å and can potentially provide information on direct versus indirect interactions and reciprocal binding sites. Gonzalez‐Lozano et al. (2020) have demonstrated the utility of this technique to reveal the architecture and assembly of synaptic protein complexes from mouse brain hippocampus. They obtained 11999 unique cross‐links corresponding to connections within and between 2362 proteins and demonstrated high fidelity and reproducibility as an experimental approach (Gonzalez‐Lozano et al. 2020). Using the AMPAR as an exemplar, and on the basis of the identified cross‐links, they were able to generate docking models for interactions of AMPAR GluA2/GluA3 heteromers with binding partners Olfactomedin 1, Ferric Chelate Reductase 1 Like, TARP γ2 and TARP γ8 (Gonzalez‐Lozano et al. 2020). A second emerging method is the use of peptide‐array technologies. Traditional peptide arrays have previously been used in GlyR and gephyrin proteomics studies for the mapping of PPI interfaces, generally by screening both binding protein partners under investigation (e.g., for GlyR α1‐sydnapin I and GlyR α1‐collybistin interactions; Langlhofer et al. 2020; Breitinger et al. 2021). These assays provide rapid qualitative or semi‐quantitative results that require validation using quantitative methods, e.g., isothermal titration calorimetry (ITC), which requires purified proteins and peptides (Schrader et al. 2004; Kim et al. 2006). However, recently a new method was described that combines array‐based peptide binding experiments with a solution assay (temperature‐related intensity change, TRIC) based on the displacement of a fluorescent marker that can be used to quantify binding affinities (Schulte et al. 2021). Proof of concept for this method was provided by mapping interactions between peptides encoding parts of the GABA_A_R α3 and GlyR β subunits and the gephyrin E domain. An overlapping peptide library consisting of 15mer peptides with an offset of one amino acid was prepared to map the benchmark interaction of the gephyrin E domain to GlyR β and GABA_A_R α3 and successfully identified critical motifs in previously known binding sites ^395^FNIVG^399^ (GABA_A_R α3) and ^420^FSIVG^424^ (GlyR β; Schulte et al. 2021). One disadvantage of this method is that it requires specific instrumentation (Dianthus) to obtain a higher signal‐to‐noise ratio compared with traditional fluorescence polarization (Iaculli and Ballet 2024).

### 9.4 Robust Systems for Functional Follow Up: Artificial Synapses

**FIGURE 4:**
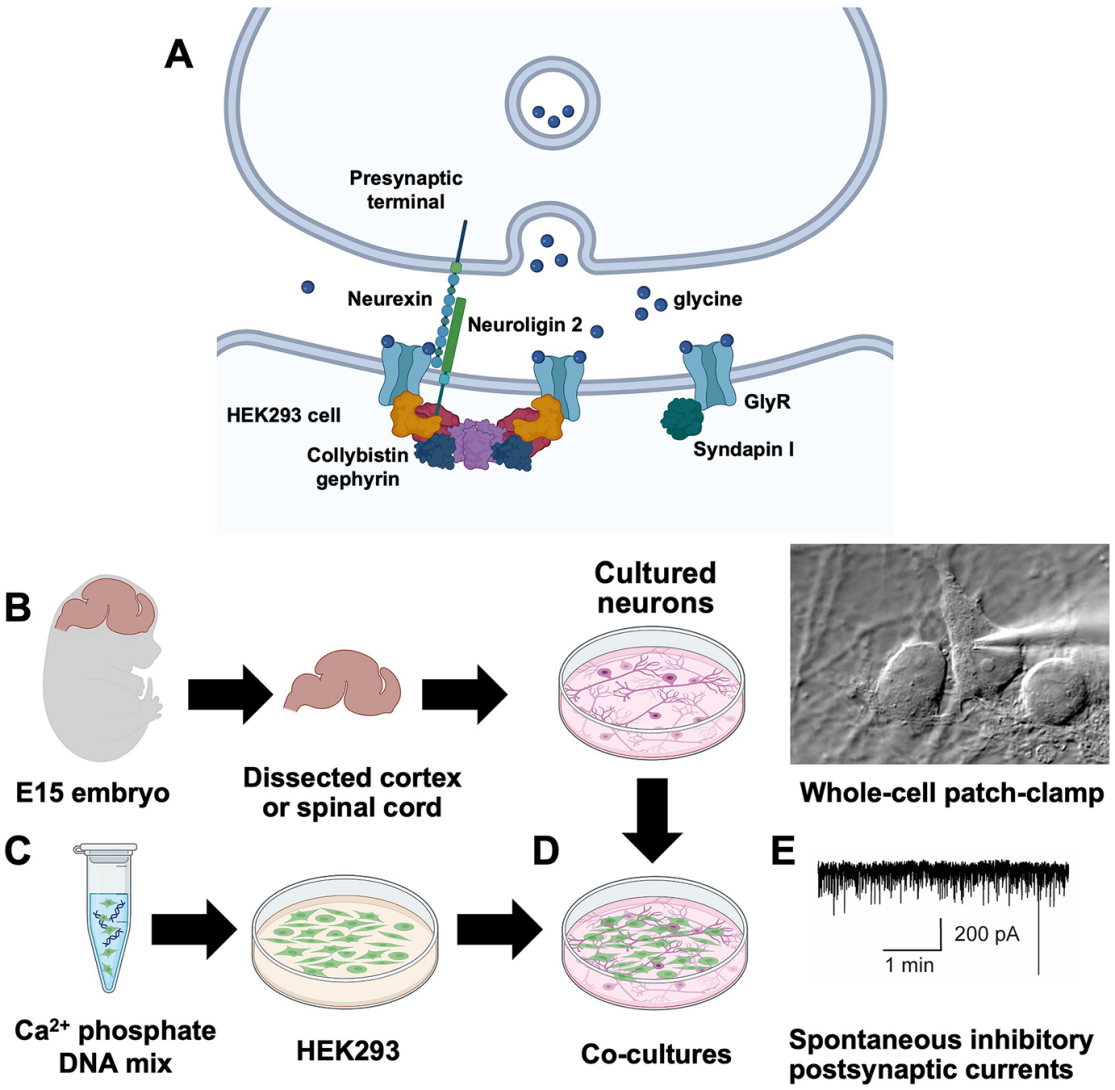
The artificial synapse system for studying GlyR interactors. (A) Artificial synapses are formed between cultured neurons (which provide presynaptic terminals) and HEK293 cells that express defined combinations of GlyR subunits (homomeric α1‐α4 or heteromeric αβ GlyRs) plus the synapse‐promoting molecule neuroligin 2. The main advantage of this system is that it allows testing of the functional properties of GlyRs under realistic synaptic activation conditions. Endogenous concentrations of glycine are released in a quantal manner by incoming presynaptic terminals, while the components of the postsynaptic HEK293 cells can be manipulated by transfection of distinct GlyR subunit combinations with or without synaptic accessory proteins, for example, gephyrin/collybistin or syndapin I. (B–E) Artificial synapses co‐culture method. (B) Neuronal cultures can be prepared from different rodent brain regions at various developmental stages. (C) HEK293 cells are then transfected with expression constructs for GlyRs, neuroligin 2, with or without accessory proteins; (D) Co‐cultures—neurons form synaptic contacts with HEK293 cells; (E) Spontaneous inhibitory postsynaptic currents (IPSCs) can be recorded from artificial synapses incorporating wild‐type GlyRs with or without accessory proteins and used to calculate mean 10%–90% rise times, IPSC decay time constants, and amplitudes. Figure created using elements from https://BioRender.com.

Successive studies using low‐throughput structural, localization, and functional assays have demonstrated physiologically relevant roles for several GlyR accessory interactors in regulating cell‐surface trafficking, synaptic localization, and channel gating kinetics (Table 1). However, while experiments using overexpression, shRNA‐mediated knockdown, high‐resolution microscopy, or interactor knockouts are the gold standard for proving functional relevance, these methods are expensive, technically complex, and time‐consuming. Before embarking on experiments in cultured neurons or knockout mice, we recommend assessing the functional impact of potential GlyR interactors in the artificial synapse system (Figure 4). Pioneered by Lynch and colleagues, this system induces the formation of artificial synapses between cultured spinal or cortical neurons and HEK293 cells expressing GlyR subunits of interest plus neuroligin 2 (Dixon et al. 2015; Zhang et al. 2015). This system recapitulates the properties of native GlyRs, since recombinant α1β and α3β GlyRs mediate fast decaying inhibitory postsynaptic currents (IPSCs) whereas α2β and α4β GlyRs mediate slow decaying IPSCs (Zhang et al. 2015; Leacock et al. 2018). Surprisingly, although this system has been used to investigate GlyR α1 and α2 mutations in startle disease and NDDs (Zhang et al. 2016, 2017; Langlhofer et al. 2020; Chen et al. 2022) this unique system has not yet been used to assess the impacts of GlyR interacting proteins such as gephyrin/collybistin or syndapin I on GlyR trafficking, localization, peak amplitude, activation, and deactivation kinetics. The major advantage of this preparation is that the effects of individual interactors can be assessed on defined GlyR subtypes in a synaptic context.

In summary, we still have much to learn about the proteomics of inhibitory GlyRs, and defining the range of GlyR accessory proteins that modulate receptor localization and functional properties will require us to embrace new methods and technologies (Figures 3 and 4), including the use of GlyR knockout lines as controls for IPs, proximity labeling, and DARPins. We also require high‐throughput methods for mapping protein–protein interactions within complexes, such as XL‐MS and quantitative peptide‐array technologies. Functional validation of interactors via overexpression/shRNA‐mediated knockdown in cell lines and cultured neurons, high‐resolution microscopy, or using interactor knockouts is vital. However, artificial synapses provide a system that can be used to triage the impacts of GlyR accessory proteins in a carefully controlled 'neuronal' context. Applying these methods to unlock the GlyR interactome will broaden our knowledge of the molecular mechanisms underlying glycinergic signaling in health and disease. Identifying new GlyR α1 and α2 accessory proteins is also likely to reveal novel candidate genes for genetic screening in startle disease and neurodevelopmental disorders, given that there are cases of startle disease that cannot be explained by mutations in known genes (encoding GlyR α1, β, GlyT2 or Asc‐1; Schaefer et al. 2022; Drehmann et al. 2023), as well as the identification of the SH3‐domain containing interactors affected by GlyR α2^P369T^ and GlyR α2^P373L^ variants (Marcogliese et al. 2022). Lastly, there is a pressing need for evidence‐based, expert‐curated knowledge that does not just encompass GlyRs and their interactors, but all synaptic proteins. In this regard, it is vital that information on synaptic PPIs is integrated into publicly available platforms such as SynGO (Synaptic Gene Ontology), an expert‐curated universal reference for synapse research and an online analysis platform for interpretation of large‐scale proteomics data (Koopmans et al. 2019; https://syngoportal.org).

## Author Contributions

**Sean D. Fraser:** investigation, methodology, validation, visualization, writing – original draft, writing – review and editing. **Remco V. Klaassen:** investigation, methodology, writing – original draft, writing – review and editing. **August B. Smit:** funding acquisition, investigation, methodology, supervision, writing – original draft, writing – review and editing. **Carmen Villmann:** funding acquisition, investigation, methodology, supervision, writing – original draft, writing – review and editing. **Robert J. Harvey:** conceptualization, data curation, formal analysis, funding acquisition, investigation, supervision, visualization, writing – original draft, writing – review and editing.

## Conflicts of Interest

The authors declare no conflicts of interest.

### Peer Review

The peer review history for this article is available at https://www.webofscience.com/api/gateway/wos/peer‐review/10.1111/jnc.70061.

## Data Availability

Data sharing not applicable to this article as no datasets were generated or analysed during the current study.
